# Impact of vocational rehabilitation on social functioning, cognitive functioning, and psychopathology in patients with chronic schizophrenia

**DOI:** 10.4103/0019-5545.44747

**Published:** 2008

**Authors:** P. N. Suresh Kumar

**Affiliations:** Govt. Medical College, Thiruvananthapuram, Kerala, India

**Keywords:** Chronic, schizophrenia, vocational, rehabilitation

## Abstract

**Aim:**

To assess the impact of vocational rehabilitation on psychopathology, social functioning and cognitive functioning in schizophrenia

**Materials and Methods:**

34 patients with DSM IV diagnosis of chronic schizophrenia were compared 40 patients with same diagnosis but not attending vocational rehabilitation using PANSS, SCARF social functioning Index and MMSE.

**Results and Discussion:**

Basic psycho-socio-demographic data were comparable in both groups except more hospitalization in the no rehabilitation group. Comparison of social functioning, cognitive functioning and psychopathology showed significant improvement in rehabilitated patients. Cognitive functioning had positive correlation with occupational role in the rehabilitated group and negative correlation in the rehabilitated group. Social functioning had negative correlation with positive and negative symptoms, general psychopathology and total PANSS score and cognitive symptoms in patients without rehabilitation.

**Conclusion:**

The present concludes that there is a definite limitation in the domains of social functioning, cognitive functioning and psychopathology in chronic schizophrenia patients who had no rehabilitation. However vocational rehabilitation significantly improves these limitations, which in turn help these patients to integrate into the society so as to function efficiently in their roles as parents, home makers and social beings.

## INTRODUCTION

Schizophrenia is one of the severe forms of mental illnesses, which demands enormous personal and economic costs. Globally it is estimated that 25 million have schizophrenia.[1] The majority of patients with schizophrenia, even those with favorable response to antipsychotics will have residual symptoms, cognitive impairments and limited social skills. This affects their ability to live in a community to the expected norms, take up employment, or to establish social relationships.

Rehabilitation is essential to help such patients to return to the previous level of functioning. It helps them to cope with the illness, strive for greater reliance and enhanced functioning and to improve the quality of life. Vocational rehabilitation programme utilizes the work for the improvement of symptoms, interpersonal relationships, and cognitive functioning. It brings forth significant changes in their over all-functioning level i.e., living, learning, and work related conditions. Vocational rehabilitation has been shown to improve employment rates for individuals with schizophrenia.[2–4] Thus, vocational rehabilitation is a central issue in the rehabilitation of patients with chronic schizophrenia.

Even though rehabilitation programmes are in progress for many years in western setting it is still under developed in our country and only limited studies have been conducted in the Indian context to evaluate their effectiveness. All these observations together with the interest in the rehabilitation of patients with chronic schizophrenia prompted the investigator to study how far vocational rehabilitation reduces the symptoms and improve social and cognitive functioning of these patients.

## OBJECTIVES

1. To identify and compare the level of social functioning, cognitive functioning, and psychopathology in patients with chronic schizophrenia undergoing rehabilitation with those patients not undergoing rehabilitation.
2. To assess the correlation between cognitive functioning, social functioning, psychopathology, and social functioning in the above two groups.

## MATERIALS AND METHODS

This study was conducted at Government Mental Health Center, Kozhikode, one of the largest state government owned mental health centers with a bed strength of 474. About 150 patients attend the outpatient department and around 10-15 patients get admitted in the family therapy ward on every day basis. This hospital has got extensive rehabilitative services. The male rehabilitation side is a well-equipped unit having facilities for school and college notebooks manufacturing, bookbinding, offset printing, carton making, medicine cover making horticulture. The female unit offers facilities for bookbinding, spinning, and medicine cover making. Both male and female rehabilitation centers work from 10.00 am to 4.00 pm with one-hour break for lunch from 1.00 pm to 2.00 pm on all government working days. Patients will be allocated appropriate job based on their aptitude and current level of functioning. These patients are paid incentives appropriate to the quality and quantity of work. On an average most of these patients are getting 75 to 100 Rupees per day.

Non-probability purposive sampling technique was used for the study. The sample was selected from the out patient population as per the criteria laid out below.

### Inclusion criteria

1. Clinically diagnosed chronic schizophrenia as per DSM IV Criteria[5] with at least two-year duration of illness.
2. Patients attending vocational rehabilitation for at least 6 months - Group I
3. Patients who had no rehabilitation during life time - Group II
4. Patients who are able to communicate in Malayalam

### Exclusion criteria

1. Associated medical illnesses severe enough for not to participate in the study
2. Seizure disorder and mental retardation
3. Acute exacerbation

### Instruments

1. Specially designed proforma for documenting socio-demographic and illness data such as age, sex, marital status, education, religion, type of family, family income, total duration of illness, duration of treatment, duration of rehabilitation and the number of hospitalization.
2. SCARF Social Functioning Index[6] was used to collect information regarding social functioning. This schedule with very good reliability and validity contains 17 questions to assess the social functioning in four major areas- self-care, occupational role, role in the family, and other social roles. It is a five point rating scale.
3. Mini Mental State examination[7] to assess the cognitive functions. Cognitive functions in the domains of orientation, registration, attention, calculation, recall, language, and construction abilities were tested. The maximum score is 30.
4. Positive and Negative Syndrome Scale[8] to assess the positive and negative symptoms and general psychopathology. This standardized tool give rating on a total of 30 symptoms, which include seven items on the positive scale, seven items on the negative scale and 16 items on the general psychopathology scale. Rating points range from one to seven (absent, minimal, mild, moderate, moderately severe, severe, and extreme). Total psychopathology score reflects the severity of illness across the parameters.

After getting approval from the institutional ethics committee data were collected during the period from 08-02-2006 to 21-03-06. A written informed consent was obtained from all the participants. Patients were comfortably seated, explanation about the objectives of the study was given and confidentiality of the data was ensured. First, the investigator interviewed the client for collecting socio-demographic data and social functioning. Following this MMSE was given and then PANSS was administered. Time taken for one client was 45 minutes.

### Data analysis

Socio-demographic data were analyzed using frequencies, percentage, and chi-square. Comparison of social and cognitive functioning and psychopathology was done by *t*-test. Correlation between cognitive functioning, social functioning, and psychopathology was performed by Pearson's Coefficient of Correlation.

## RESULTS

Tables 1 and 2 show the comparison of psycho-socio-demographic characteristics patients who are attending and not attending vocational rehabilitation. Mean age, gender, education, type of family, family history of mental illness, family support, duration of treatment, and duration of illness were comparable in both groups. Muslims, those with monthly family income between 1501 and 3000, those with at least one hospitalization within one year, and married were significantly higher in the no rehabilitation group.

**Table 1:** Comparison of demographic characteristics of patients with and without vocational rehabilitation

	Rehabilitation *N*=34 (%)	No rehabilitation *N*=40 (%)	X^2^/t	*P*
Mean age	37.88	37.48		
Sex				
Male	25 (73.5)	29 (72.5)	0.92	0.56
Female	9 (9)	11 (27.5)		
Education				
Illiterate	0 (0)	2 (5)	4.39	0.49
Primary	5 (44.1)	16 (40)		
High school	11 (32.4)	16 (40)		
Secondary	2 (5.9)	3 (7.5)		
College	6 (17.6)	3 (7.5)		
Religion				
Hindu	30 (88.2)	17 (42.5)	20.78	0.00
Christian	2 (5.9)	1 (2.5)		
Muslim	2 (5.9)	22 (55)		
Monthly family income (Rs)				
<1500	29 (85.1)	29 (72.5)	8.80	0.01
1501-3000	2 (5.9)	11 (27.5)		
3001-4000	3 (8.8)	0 (0)		
Marital status				
Unmarried	17 (50)	20 (50)	9.22	0.05
Married	35 (5)	14 (35)		
Widow/widower	0(0)	1 (2.5)		
Separated /divorced	12 (35.3)	5 (12.5)		
Type of family				
Nuclear	20 (58.8)	26 (65)	0.29	0.58
Extended	14 (41.2)	14 (35)		

Figures in parentheses are in percentage

**Table 2:** Comparison of illness variables of patients with and without vocational rehabilitation

	Rehabilitation *N*=34 (%)	No rehabilitation *N*=40 (%)	X^2^/t	*P*
No. of hospitalization within 1 year				
Nil	23 (67.6)	10 (25)	7.04	0.01
Once	6 (17.7)	29 (72.5)		
Twice	3 (8.8)	0 (0)		
>Twice	2 (5.9)	1 (2.5)		
Family H/O of mental illness	14 (41.2)	12 (30)	1.0	0.31
Family support	25 (73.5)	25 (62.5)	4.04	0.13
Duration of illness				
1-3 years	4 (11.8)	5 (12.5)	5.33	0.14
4-6 years	9 (26.4)	5 (12.5)		
7-10 years	2 (22.5)	9 (22.5)		
>10 years	19 (52.5)	21 (52.5)		
Duration of treatment				
<1 year	2 (5.9)	0 (0)	3.47	0.32
1-5 years	12 (35.3)	12 (30)		
6-10 years	5 (14.7)	10 (25)		
>10 years	15 (44.1)	18 (45)		

Figures in parentheses are in percentage

Table 3 shows the comparison of social functioning, cognitive functioning, and psychopathology of patients with and without vocational rehabilitation. Social functioning domains - self care, occupational role, role in family, social role and total score and cognitive domains - orientation, attention, language and total MMSE score were significantly higher in patients with rehabilitation. In the psychopathology domain scores of positive and negative syndrome, general psychopathology, anergia, thought disturbance and paranoid were significantly lower in patients with rehabilitation.

**Table 3:** Comparison of social functioning, cognitive functioning, and psychopathology in patients with and without vocational rehabilitation

	Rehabilitation Mean ± SD	No rehabilitation Mean ± SD	X^2^/t	*P*
Social functioning				
Self care	21.8±1.07	17.50±3.50	6.62	0.000
Occupational role	13.5±1.19	7.58±2.81	11.57	0.000
Role in the family	16.32±3.15	13.85±2.75	3.61	0.001
Social role	17.97±1.87	14.53±2.96	5.863	0.000
Total social	69.74±4.38	53.45±9.23	9.423	0.000
functioning				
Cognitive functioning				
Orientation	9.26±1.42	7.30±2.45	−4.11	0.000
Registration	2.94±0.24	2.70±0.65	−2.05	0.440
Attention	4.09±1.16	2.95±1.72	−3.267	0.002
Recall	2.26±0.75	1.90±0.93	−1.836	0.070
Language	7.35±0.69	6.73±0.96	−3.176	0.002
Total MMSE score	26.59±2.99	22.08±5.48	−4.286	0.000
Psychopathology				
Positive syndrome	8.68±1.61	12.68±5.08	4.401	0.000
Negative syndrome	7.71±1.29	14.13±7.73	4.781	0.000
General	17.29±1.29	24.43±11.63	3.553	0.001
psychopathology				
Anergia	4.44±0.96	6.70±4.54	2.844	0.006
Thought disturbance	4.65±0.85	5.75±2.07	2.901	0.005
Activation	3.24±0.43	3.58±1.58	1.212	0.299
Paranoid	3.56±0.66	5.90±3.64	3.699	0.000
Depression	4.15±0.36	4.75±1.78	1.940	0.056

Table 4 shows the correlation analysis of social functioning with cognitive functioning and psychopathology. Cognitive functioning had positive correlation with occupational role in the rehabilitated patients and negative correlation in the non-rehabilitated patients. Cognitive symptoms had negative correlation with total score of social functioning in non-rehabilitated patients. Social functioning had negative correlation with positive and negative syndrome, general psychopathology and total PANSS score in patients without rehabilitation.

**Table 4:** Correlation analysis of social functioning with cognitive functioning and psychopathology

	Rehabilitation *r*	No rehabilitation *r*
Cognitive functioning		
Social functioning		
Occupational role	0.349*	0.297
Social role	0.151	−0.323*
Total social functioning score	0.236	−0.453*
Social functioning		
Psychopathology		
Positive syndrome	−0.183	−0488*
Negative syndrome	−0.214	−0.636*
General psychopathology	−0.296	−0.697*
Total symptoms	0.140	−0.640**

* Correlation is significant at 0.01 level,

** Correlation is significant at 0.01 level

## DISCUSSION

The present study proves that vocational rehabilitation for six months improves social functioning, reduces the severity of symptoms, reduces re-hospitalizations and enhances cognitive functioning in patients with chronic schizophrenia. This finding is in concordance with the findings of many western studies.[4,9–13]

Vocational rehabilitation showed significant improvement in positive symptoms, negative symptoms, thought disturbance and paranoid ideation. Mueser *et al*.[14] in their study on work and non-vocational domains of function found that patients who are working showed lesser symptoms, particularly in thought disorder and a higher global assessment score. Thara and Sreenivasan[15] have found significant improvement in the domains of under activity, social withdrawal, participation in the family, and work performance in patients undergoing vocational rehabilitation. Ajimol[16] from Bangalore have noted a higher degree of over all functioning and reduced symptoms in rehabilitated patients compared to those not vocationally rehabilitated.

In schizophrenia, neurocognitive factors play an important role in respect of their social competence. Present study revealed a positive correlation between social functioning especially occupational role and cognitive functioning in patients who had undergone vocational rehabilitation. Same time patients without vocational rehabilitation had negative correlation of social functioning with cognitive functioning. These findings are in consistent with the findings of Bryson and Bell,[17] Liddle,[18] and Penn and Mueser.[19] Harvey *et al.*[20] found that cognitive functioning is a good predictor of over all functioning.

Present study showed significant negative correlation of social functioning with positive symptoms, negative symptoms, and general psychopathology in patients with vocational rehabilitation. This is also in accordance with the findings of Mc Gurk and Mueser[21] and Hoffman and Kupper.[22] It is obvious that negative symptoms are associated with deterioration in work function. To substantiate this point negative symptoms were significantly less in patients who had vocational rehabilitation. Probably low-level of negative symptoms secondary to vocational rehabilitation might have helped these patients to function better in various social domains. Laroche[23] and Lysaker and Bell[24] have also observed poor social functioning among patients with a high level of negative symptoms. Srinivasan and Tirupati[25] found that disability in work performance is correlated with mean scores on PANSS and general psychopathology scale. Kurtz and Moberg[26] also reported that symptoms like psychomotor poverty, disorganization and cognitive decline are related to social functioning.

Before concluding, some of the methodological limitations of this study have to be considered. Being a smaller sample and cross sectional study with only one point assessment without baseline assessment prior to rehabilitation generalization of our findings is limited. Investigator was not blind in assessing patients with and without rehabilitation. Moreover, the investigator could not control extraneous factors like physical and psychological factors and medications, which may influence the studied variables.

The present study draws the conclusion that there is a definite limitation in the domains of social functioning, cognitive functioning and psychopathology in chronic schizophrenia patients who had no rehabilitation in their life time. However, vocational rehabilitation significantly improves these limitations, which in turn help these patients to integrate into the society so as to function efficiently in their roles as parents, home makers, and social beings. Being the only source of income in many patients, vocational rehabilitation has significantly helped to earn livelihood for buying medicines as well as to look after the family. Vocational rehabilitation has also reduced the relapse rate and subsequent hospitalization which indirectly reduces the treatment cost and burden for the caregivers. An attractive feature of this type of rehabilitation is that all patients irrespective of their functional status can be accommodated to different types of rehabilitative work available in the local community. Considering the cost effectiveness and lower cost in implementation, this type of rehabilitation may be an ideal model for a developing country like India. In this context, there is a need for future studies with larger sample size and with more longitudinal and periodical assessments to assess the impact of vocational rehabilitation using locally available means.
